# A porcine model of acute, haematogenous, localized osteomyelitis due to *Staphylococcus aureus*: a pathomorphological study

**DOI:** 10.1111/j.1600-0463.2010.02700.x

**Published:** 2011-02

**Authors:** Louise Kruse Johansen, Dorte Frees, Bent Aalbæk, Janne Koch, Tine Iburg, Ole Lerberg Nielsen, Pall Skuli Leifsson, Henrik Elvang Jensen

**Affiliations:** Department of Veterinary Disease Biology, Faculty of Life Sciences, University of CopenhagenFrederiksberg, Denmark

**Keywords:** Haematogenous osteomyelitis, *Staphylococcus aureus*, pig, intra-arterial inoculation, pathomorphology

## Abstract

A porcine model of acute, haematogenous, localized osteomyelitis was established. Serial dilutions of *Staphylococcus aureus* [5–50–500–5000–50 000 CFU/kg body weight (BW) suspended in saline or saline alone] were inoculated into the right brachial artery of pigs (BW 15 kg) separated into six groups of two animals. During the infection, blood was collected for cultivation, and after the animals were killed from day 5 to 15, they were necropsied and tissues were sampled for histopathology. Animals receiving ≤500 CFU/kg BW were free of lesions. Pigs inoculated with 5000 and 50 000 CFU/kg BW only developed microabscesses in bones of the infected legs. In the centre of microabscesses, *S. aureus* was regularly demonstrated together with necrotic neutrophils. Often, bone lesions resulted in trabecular osteonecrosis. The present localized model of acute haematogenous osteomyelitis revealed a pattern of development and presence of lesions similar to the situation in children. Therefore, this model should be reliably applied in studies of this disease with respect to e.g. pathophysiology and pathomorphology. Moreover, because of the regional containment of the infection to a defined number of bones, the model should be applicable also for screening of new therapy strategies.

Haematogenous osteomyelitis (HO) especially caused by *Staphylococcus aureus* is often seen in prepubertal children and has a propensity for localization in the metaphyseal area of long bones (1, 2). Early diagnosis and treatment of HO are difficult. Therefore, lesions often result in long-term morbidity and severe complications such as pathological fractures, arthritis, growth arrest and bone deformity (3, 4). Moreover, an increasing number of cases are caused by methicillin-resistant *S. aureus*, which have put further focus on the risk of a negative outcome of therapy (5, 6).

Several animal models have been developed aiming to evaluate the pathogenesis, diagnosis and treatment of bone infections caused by *S. aureus* (7). However, the vast majority of the models are based on inoculation of bacteria directly into the bone marrow together with a sclerosing or foreign body material (bone wax or polymethylmethacryl), to promote the inflammatory response (7). However, these models do not meet the requirements of a discriminative animal model for HO in which the port of entry should be by blood-borne bacteria (8).

Therefore, models based on intra-arterial inoculation and subsequent development of local osteomyelitis might be the most reliable models for extrapolation to paediatric cases of HO. Inoculation in the arterial supply to selected bones should be advantageous compared with systemic intravenous inoculation because of the localized approach, which constitutes a relevant model for naturally occurring HO in single bones (9). Intra-arterial inoculation has only been used in a canine model, and although HO successfully was established, severe problems with sepsis were apparent (10).

Optimally, a discriminative model for HO should be established in an animal species that mimics the physiological profile of humans. The size of the pig and its anatomy, nutrient requirement, metabolic rate, general physiological behaviour, pattern of organ development and capillary density make it useful as a model for many human diseases, including HO (8). An advantage comes from the fact that *S. aureus* is a frequent cause of spontaneous osteomyelitis in pigs with similarities in pathogenesis and pathology to the situation in children (11, 12). Curiously, only three porcine models of osteomyelitis have been described (11, 13, 14). Two of the models were based on traumatic intramedullar inoculation techniques (13, 14) and one on systemic intravenous inoculation (11). Although chronic osteomyelitis was successfully developed in the two traumatic models, problems with wound contamination were apparent (13, 14). In the model based on intravenous inoculation, acute osteomyelitis developed in the metaphyseal area of especially the long bones of the limbs, but the animals also developed embolic pneumonia and sepsis (11, 15).

The aim of the present study was to develop a porcine model for localized HO by inoculation of *S. aureus* into the right brachial artery. Different doses of *S. aureus* were used to determine the optimal inoculation count causing HO while avoiding systemic spread of the bacterium.

## Materials and Methods

### Animals

Twelve healthy female Yorkshire-Landrace crossbred pigs, with a BW of approximately 15 kg (age 8–9 weeks), obtained from a specific pathogen-free herd were used (16). At arrival, the animals were allowed to acclimatize for 7 days before entering the trial. The animals were fed a commercial pig diet (Svine Erantis Brogaarden ApS, Lynge, Denmark) *ad libitum* and had free access to tap water. Before entering the experiment, the animals underwent a clinical examination.

### Bacterial strain and preparation of inocula

*Staphylococcus aureus* strain S54F9, originally isolated from a chronic embolic porcine lung abscess and previously used for experimental inoculation of pigs was used (11, 15, 17). The strain was propagated for 18 h at 37 °C in Luria–Bertani (LB) broth (18) with shaking, sedimented by centrifugation at 3000 *g* for 30 min and resuspended in sterile isotonic saline. The viable count was determined by the plate count method (18), and the suspension was diluted with sterile isotonic saline 0.9% to obtain dilutions of 5–50–500–5000–50 000 CFU/kg BW in a volume of 0.5 mL.

### Experimental procedure and groups

**Fig. 1:**
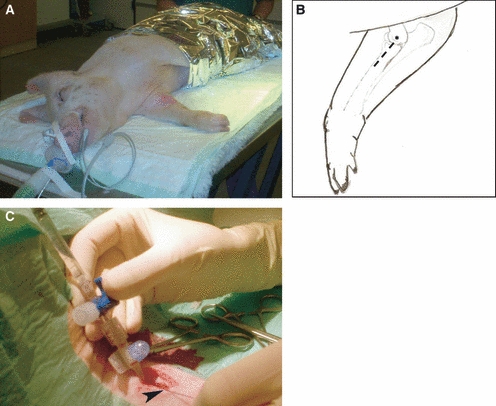
The inoculation procedure. (A) Anaesthetized pig in right lateral recumbency with the right medial ante-brachium prepared for surgery. (B) Schematic drawing, the skin incision is indicated by the punctured line and the asterisk (*) shows the location of the bone protuberance *epicondylus medialis humeri*. (C) Inoculation through a catheter equipped with a three-way stopcock inserted into the right brachial artery fixated with a ligature (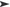).

The animals, which remained clinically healthy during the time of acclimatization, were randomly assigned into six groups (A–F) of two pigs, each receiving one of the five doses. One group received a placebo inoculation with sterile saline. An overview of the design is given in Table 1. After sedation as described recently (17), the pigs were placed in right lateral recumbency. Flexing of the left front leg made the medial right ante-brachium available for surgery (Fig. 1A). Sterile conditions were maintained during the entire inoculation procedure. A skin incision (3 cm) starting at *epicondylus medialis humeri* and going distally parallel to the radial bone was made (Fig. 1B) making the *pronator teres* and *flexor carpi radialis* muscles visible. Separation of the muscles led to accessibility to the brachial artery and vein. *Arteria brachialis* (*a. brachialis*) was purified and isolated from the surrounding tissue, and two loose ligatures (Vicryl 4/0 ETHICON; Johnson & Johnson Company, St-Stevens-Woluwe, Belgium) were placed around the artery. The artery was ligated with the proximal ligature and a catheter (Optiva I.V. Catheter 22G; Smiths Medical, Milano, Italy) equipped with a three-way stopcock (Discofix 3-way stopcock, B. Braun, Melsungen, Germany) was guided a few centimetres into the artery in the distal direction. Inoculation was performed through the catheter which afterwards was flushed with 5 mL of sterile saline (Fig. 1C). While carefully removing the catheter, the distal ligature was tightened. The subcutaneous tissue was sutured in a continuous pattern (Vicryl plus 4/0 ETHICON, Johnson & Johnson Company), and the skin by four sutures in an interrupted pattern (Vicryl plus 4/0 ETHICON, Johnson & Johnson Company). After surgery, the animals lived for 5–15 days whereupon they were killed by an overdose of 20% pentobarbital given intravenously (Table 1). Throughout the experimental period, lame animals received intramuscular injections of buprenorfin (Temgesic 0.3 mg/mL; Schering-Plough, Heist-op-den-Berg, Belgium) every sixth to eighth hour.

**Table 1:** Experimental protocol and the histopathological scores of intraosseous acute inflammation in bones

Groups	Animal no.	Dose	Time from inoculation to euthan-asia	IAI scores in selected bones of the right front leg									
				
		CFU/kg BW	Days	Radius	Ulna	Os metacarpale III	Os metacarpale IV	Phalanx proximalis III	Phalanx proximalis IV	Phalanx medialis III	Phalanx medialis IV	Phalanx distalis III	Phalanx distalis IV
A	1	0	15	0	0	0	0	0	0	0	0	0	0
	2	0	15	0	0	0	0	0	0	0	0	0	0
B	3	5	14	0	0	0	0	0	0	0	0	0	0
	4	5	14	0	0	0	0	0	0	0	0	0	0
C	5	50	08*,**	0	0	0	0	0	0	0	0	0	0
	6	50	14	0	0	0	0	0	0	0	0	0	0
D	7	500	08*,**	0	0	0	0	1	0	0	0	0	0
	8	500	13	0	0	1	1	0	0	0	0	0	0
E	9	5000	13	1	1	1	0	1	0	0	0	0	0
	10	5000	05*	1	1	2	0	2	1	4	1	4	1
F	11	50 000	05*,**	4	1	0	3	4	0	1	0	0	0
	12	50 000	05*	0	0	4	1	1	0	0	0	0	0

IAI, intraosseous acute inflammation.

The scores of IAI are reported with Arabic numbers (0–4): 0, Not present; 1, Minimal to mild inflammation with no intramedullary abscess; 2, Moderate to severe inflammation with no intramedullary abscess; 3, Minimal to mild inflammation with intramedullary abscess; 4, Moderate to severe inflammation with intramedullary abscess.

* Animals killed due to lameness.

** Phlegmon formation.

The Danish Animal Experimental Act approved the protocol (licence no. 2008/561-37).

### Blood samples

Screening for bacteraemia included bacteriological examination on whole blood. The blood samples were taken from the jugular vein 5 days prior to inoculation and approximately 7 and 14 days after challenge. Blood from pigs killed earlier than day 7 were taken just before the animal was euthanized. The blood samples were collected in 10-mL heparin tubes (NH170 I.U. BD Vacutainer, Playmouth, UK) and kept at 5 °C for a maximum of 4 h until processing. Blood samples in volumes of 1 mL and 1 mL of decimal dilutions (using sterile isotonic saline) were added to empty Petri dishes and mixed with melted LB agar medium. Viable count was determined after incubation for 48 h at 37 °C and presented as counts/mL blood.

### Pathology

After killing, all animals were necropsied, and sampling of tissues for histopathology included specimens of the lung taken from the dorsal margo of the left diaphragmatic lobe. Specimens of the distal growth-plate areas of the right and left radial and ulnar bones were sampled using an oscillating saw (IM-MAX MEDICAL, Frederiksberg, Denmark). Because of the fragility and small size of the bones in the distal part of the forelimbs, they were fixed *in toto*. All tissues were immersion fixed in 10% neutral buffered formalin for 3 days. After fixation, the osseous tissues were decalcified in a solution containing 3.3% formaldehyde and 17% formic acid for 2 weeks. After fixation, soft tissues and the distal growth-plate area of radial and ulnar bones, the third and fourth metacarpal bones and the third and fourth phalanges from both the left and right sides were processed through graded concentrations of alcohols and xylene, and embedded in paraffin wax. Sections of 4–5 μm were cut and stained with haematoxylin and eosin (HE) and in selected cases by phosphotungstic acid haematoxylin (PTAH) for demonstration of fibrin (19).

### Immunohistochemistry

For immunostaining, 4–5 μm tissue sections were mounted on adhesive glass slides (Thermo Scientific, Menzel GmbH & CoKG, Baunschweig, Germany). An indirect immunostaining technique based on a specific *S. aureus* murine monoclonal antibody (ab37644; Abcam plc, Cambridge, UK) was used for the *in situ* identification of *S. aureus*. The immunostaining was performed by application of the UltraVision LP Detection System HRP (Lab Vision Corporation, Fermont, CA, USA). Briefly, after deparaffinization, antigen retrieval was carried out by treatment with 0.1% trypsin (Sigma-Aldrich Denmark A/S, Vallensbæk Strand, Denmark) solution for 15 min at 37 °C. This was followed by blocking of endogenous peroxidase activity by 0.6% H_2_O_2_ for 15 min and blocking of unspecific binding by Ultra V Block (Lab Vision Corporation). The horseradish peroxidase (HRP) polymer was added and the reaction was developed with AEC (amino-ethyl-carbazol) Single Solution as described by the manufacturer (Lab Vision Corporation). Throughout the immunostaining protocol, with the exception of the step between blocking of unspecific binding and the application of the primary antibody, slides were washed in Tris-buffered saline, pH 7.6. After immunostaining, the sections were counterstained with Mayer’s haematoxylin. Negative controls were run on parallel sections and included deletion of the primary antibody and substitution of this with a nonsense monoclonal (matching isotype) antibody of same concentration as that of the primary antibody.

### Assessment criteria

Histopathological grading of inflammation was assessed based on intraosseous acute inflammation (IAI), intraosseous chronic inflammation (ICI), periosteal inflammation (PI) and bone necrosis (BN) according to the system of Smeltzer et al (20). The adopted scoring system with histopathological grading pattern of IAI and BN is given in Table 2.

**Table 2:** Histological parameters and scoring system of intraosseous acute inflammation and bone necrosis [Smeltzer et al. (20)]

Intraosseous acute inflammation (IAI)
0:Not present
1:Minimal to mild inflammation with no intramedullary abscess
2:Moderate to severe inflammation with no intramedullary abscess
3:Minimal to mild inflammation with intramedullary abscess
4:Moderate to severe inflammation with intramedullary abscess
Bone necrosis (BN)
0:No evidence of necrosis
1:Single focus of necrosis without sequestrum formation
2:Multiple foci of necrosis without sequestrum formation
3:Single focus of sequestrum
4:Multiple foci of sequestra

## Results and Discussion

**Fig. 2:**
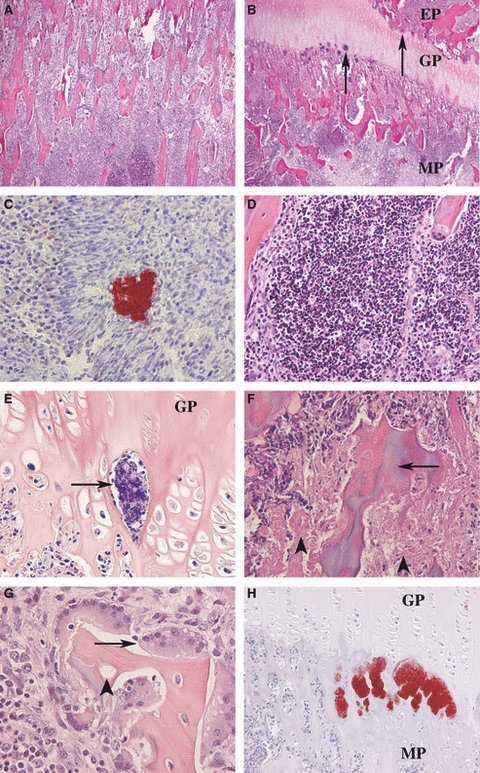
Histopathology in pigs inoculated intra-arterially (a. brachialis dextra) with 50 000 CFU/kg BW. (A) Radius: a microabscess located deep in the metaphyseal area, H&E. (B) Metacarpale III: colonies of bacteria (→) are present at the junction between the growth plate (GP) and the metaphysis (MP) and the epiphysis (EP), H&E. (C) Epiphysis of phalanx proximalis III: multiple *Staphylococcus aureus* bacteria are seen centrally in the microabscess. Surrounding cells are arranged in a pattern of palisades. Immunostaining for *S. aureus*. (D) The centre of the microabscesses was made up by accumulation of neutrophils, H&E. (E) Within the blood vessels of the growth plate (GP), fibrin deposition was sometimes observed (→), phosphotungstic acid haematoxylin (PTAH). (F) Osteonecrosis (→) was often present just beneath the growth plate together with areas of necrotic bone marrow cells (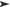), H&E. (G) Trabeculae with empty lacunae (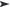) were typically surrounded by bone resorbing osteoclasts (→), H&E. (H) Multiple *S. aureus* bacteria were often identified in connection with the capillary loops at the junction between the growth plate (GP) and the metaphysis (MP). Immunostaining for *S. aureus.*

Intra-arterial inoculation into *a. brachialis* dexter of pigs with *S. aureus* results in the development of osteomyelitis lesions in different bones supplied by the artery, whereas lesions were not observed in the control animals (Table 1). Bacteria and accompanied inflammatory reaction were localized deep in the metaphysis, next to the cartilage of the growth plate, or next to the resting zone of the growth plate, i.e. in the epiphysis (Fig. 2A–C). HO is most common in prepubertal children where the lesions typically initiate adjacent to the growth plate (1, 9). Occasionally, children with HO show acute signs of infection including fever, irritability, lethargy and local signs of inflammation (2). These clinical manifestations of paediatric osteomyelitis parallel the pattern in the present porcine model of acute stages of HO as five pigs were killed on day five or eight because of lameness of the infected leg (Table 1). Pigs killed 5 days after challenge displayed clinical signs of inflammation characterized by fever, i.e. a rectal temperature above 39.5 °C, and by oedema, redness and heat of the infected leg. Wound infections with phlegmon formation were apparent in three of the pigs (Table 1). Both pigs in the control group remained healthy throughout the trial.

Acute osteomyelitis lesions (microabscesses) were present in the two groups receiving an inoculum of 5000 and 50 000 CFU/kg BW, respectively (Table 1). In addition, pigs inoculated with 50 000 CFU/kg BW developed a suppurative arthritis in the carpal and metacarpophalangeal joints, which sometimes also is seen secondarily to HO in children (4). In group D, IAI characterized by a mild infiltration dominated by neutrophils were giving an IAI score of 1. In groups E and F, IAI was present in different bones and the scorings ranged from 1 to 4. The number of bones evaluated as score 4 increased in group F. None of the animals developed ICI or PI. The microabscesses primarily were made up by neutrophils and mononuclear cells (Fig. 2D), some with elongated cytoplasm arranged in a pattern of surrounding palisades (Fig. 2C). Regularly, oedema and haemorrhage were observed together with the microabscesses in groups E and F. Along with the influx of neutrophils in the area of capillary loops of the metaphysis, thrombosis formation was sometimes demonstrated by the deposition of fibrin (Fig. 2E). The centre of microabscesses given an IAI score of 4 occasionally showed mild BN up to score 2 (Fig. 2F). Decreased volumes of bone trabeculae surrounded by high numbers of osteoclasts and with the presence of empty lacunae were observed in relation to the lesions in both groups E and F (Fig. 2G). Devitalization of bone tissue and increased activation of osteoclasts are important events in the pathogenesis of osteomyelitis (2). The immunohistochemical reactivity was in agreement with the *in situ* presence of bacteria. Bacteria, both as single organisms and in colonies, were found in both groups E and F. The bacteria were primarily located within the centre of the microabscesses and within and around the capillary loops beneath the growth plate (Fig. 2C,H). In general, an IAI score of 4 was consistent with marked immunoreactions.

Haematogenous osteomyelitis develops after an episode of bacteraemia (2, 9). Therefore, animal models based on inoculation directly into the blood are most relevant with reference to the pathogenesis. Animal models of HO based on intravenous inoculation have been established, but problems with localization in other organs, especially the lungs, are hampering these models (7, 11, 21, 22). Intra-arterial inoculation of *S. aureus* for the development of HO has only been described in a canine model (10). In this canine model, 0.1 mL of a culture of *S. aureus* (10^6^ CFU/mL) was inoculated into the nutrient artery of the tibial bone. However, in the model, lesions also were seen systemically. By contrast, no signs of systemic spread were observed in any of the intra-arterially inoculated pigs in the present study as documented by sterile blood samples and the containment of lesions to the infected leg. An explanation for this difference might be the smaller amount of inoculum used and the presence of pulmonary intravascular macrophages in pigs, which promptly eliminate haematogenously spread *S. aureus* bacteria (23).

The relationship between inoculation doses and histopathological changes in porcine models of osteomyelitis or in models inoculated intra-arterially has not been examined. In the present model, the development of suppurative bone lesions required a bacterial density of 5000 CFU/kg BW. In comparison, a dose–response study was established in a well-defined chicken model based on intravenous inoculation, where the development of acute HO in 50% of the animals required 10^5^CFU/kg BW (24). With reference to the three Rs pronounced by Russel and Burch in 1959 (25), the present porcine HO model is fulfilling the expectation of refinement and reduction because of its discriminative nature, containment of lesions and the predictive occurrence of lesions compared with established haematogenously induced HO models (11, 21, 22).

*Staphylococcus aureus* is the major aetiological agent of osteomyelitis (9). In recent years, the number of human cases caused by antibiotic-resistant *S. aureus* has increased leading to longer and more severe disease courses (5, 6). The bacterium has a strong ability to colonize bone tissue, especially at the point of endocondral ossification (26). In accordance, huge amounts of *S. aureus* were identified in this area by the immunohistochemical staining procedure.

Osteomyelitis is frequently diagnosed in slaughter pigs and shares similarities in pathogenesis and pathology with lesions in children (11, 12). In the present study, we have shown that intra-arterial inoculation of pigs is feasible and resulted in localized osteomyelitis lesions comparable to those seen in children. An inoculum dose between 5000 and 50 000 CFU/kg BW should be preferred for further studies of HO in the porcine model.
